# Exploring the association between STOX1:p.(Tyr153His) variant and preeclampsia risk in Egyptian women

**DOI:** 10.1038/s41598-025-20238-9

**Published:** 2025-09-18

**Authors:** Amira Kotb, Sandra Wagdy, Heba Sharaf, Mona Sediek, Hala Ashraf

**Affiliations:** 1 Clinical and Chemical pathology department, Faculty of Medicine, Cairo University, Cairo, Egypt; 2 Obstetrics and Gynecology department, Faculty of Medicine, Cairo University, Cairo, Egypt

**Keywords:** Pregnancy, Preeclampsia, Hypertension, Genetic etiology, *STOX1* gene, Rs1341667, Genetics, Risk factors, Reproductive disorders

## Abstract

Preeclampsia is a multi-factorial disease, with both genetic and environmental factors contributing to its development. The genetic susceptibility in preeclampsia has been determined to be around 50%. STORKHEAD_BOX1 PROTEIN 1 (*STOX1*), is the gene of interest in this study. The most frequent variant of this gene is c.457T > C (rs1341667). This case-control study was conducted on 96 participants recruited from both the Obstetrics outpatient clinic at Kasr Al Ainy hospital and the High-Risk Pregnancy Department, Cairo University. Patients were divided into 2 groups: (group I: 48 pregnant females with preeclampsia diagnosed on basis of the American College of obstetrics and gynecology criteria, group II: 48 healthy control pregnant females of matching age were included. After collecting the blood sample, DNA was extracted and detection of STOX1(NM_001130161.3): c.457T > C: p. (Tyr153His) gene variant by TaqMan Real-Time PCR were done on all involved individuals. The homozygous CC genotype, previously linked to increased preeclampsia risk, was identified in 27.1% of controls (*n* = 13) and 31.3% of cases (*n* = 15), with no statistically significant difference (*P* = 0.654). The heterozygous CT genotype, associated with moderate risk, was observed in 41.7% of controls (*n* = 20) and 39.6% of cases (*n* = 19) (*P* = 0.835). The inheritance model analysis showed no statistically significant association between the STOX1 c.457T > C variant and preeclampsia under any of the tested models. Genotypic distribution conformed to Hardy-Weinberg equilibrium in both groups, supporting the absence of deviation. These findings suggest no significant association between the STOX1 (NM_001130161.3): c.457T > C (p.Tyr153His) variant and susceptibility to preeclampsia in the studied population.

**Supplementary Information:**

The online version contains supplementary material available at 10.1038/s41598-025-20238-9.

## Introduction

Preeclampsia (PE) is among the most prevalent and dangerous pregnancy problems. The trio of elevated blood pressure, proteinuria, and edema following the 20th week of pregnancy are its defining characteristics^5^. Genetic and environmental variables both play a role in the development of preeclampsia, making it a multifactorial disease^1^. Distinguishing the individual contributions of maternal and fetal genetics from environmental influences has been challenging. A large population-based study using Swedish Birth and Multi-Generation Registries, encompassing over 700,000 pregnancies from more than 240,000 sibling pairs, estimated that 35% of the variance in preeclampsia risk is attributable to maternal genetic factors, 20% to fetal genetic factors, and 13% to a couple specific effect, potentially reflecting maternal–paternal genetic interactions. Shared familial environment contributed less than 1% to the variance, indicating a minimal role of common environmental factors, while 32% remained unexplained. These findings suggest that genetic factors, particularly maternal, account for more than half of the overall risk for preeclampsia^2^.

The fundamental pathophysiology of PE is failure of utero-vascular change. Numerous single nucleotide variations in important regulatory elements that might compromise this process have been discovered in the last 15 years as preeclampsia’s genetics have come under more evaluation. Initially, STORKHEAD_BOX1 PROTEIN 1 (*STOX1*) was recognized as a potential gene with an increased risk of developing preeclampsia. The most common variation of this gene is c.457T > C (rs1341667), which causes the amino acid in the DNA binding domain of the STOX1 protein to shift from tyrosine to histidine (Y153H)^3^, this amino acid substitution impairs trophoblast invasion by upregulating α-T-catenin (CTNNA3), which disrupts the epithelial-to-mesenchymal transition of extravillous trophoblasts^6^. Functional studies also indicate that the Y153H mutation leads to dysregulation of multiple downstream targets, including altered transcriptional activity mediated via interaction with E2F3, and widespread gene expression changes in trophoblast models^3^. Consequently, this variant impacts several pathways essential for proper placental development and vascular remodeling.

The findings of certain investigations offered more proof of *STOX1* involvement in the pathophysiology of PE, even if it was categorized as a benign variant^4^. In a cohort of 500 pregnant Turkish women, the STOX1 c.457T > C (p.Tyr153His) variant was found to be significantly associated with an increased risk of early-onset preeclampsia^5^. Given that genetic factors are estimated to contribute to approximately 50% of preeclampsia cases, and with the current prevalence of hypertensive disorders of pregnancy in Egypt reported at around 4% (EJCM), there is a growing need for routine genetic screening, particularly in women of reproductive age or during prenatal care. This study aims to investigate the potential association between the STOX1:p.(Tyr153His) variant (rs1341667) and the development of preeclampsia among pregnant Egyptian women.

## Materials and methods

### Research design

This case-control study included 96 Egyptian pregnant women recruited from the Obstetrics Outpatient Clinic and the High-Risk Pregnancy Unit at Kasr Al Ainy Hospital, Cairo University. Participants were equally divided into two groups (48 in each):

- Group I (Case group): Pregnant women diagnosed with preeclampsia according to the American College of Obstetricians and Gynecologists (ACOG) criteria (Rana et al., 2019).
- Group II (Control group): Normotensive pregnant women.

All participants were aged between 20 and 35 years, had singleton pregnancies, and were between 20 and 37 weeks of gestation. Exclusion criteria included chronic hypertension, diabetes mellitus, renal disease, other causes of proteinuria, and risk-enhancing conditions such as twin pregnancies. Demographic and clinical data were collected, including maternal age, gestational age, body mass index (BMI), medical history, family history of preeclampsia, and onset of preeclampsia (for case group), along with relevant laboratory findings (see Supplementary Tables 1 and 2). The study protocol was approved by the Research Ethics Committee (REC) of the Faculty of Medicine, Cairo University (Approval No. MS-449-2022). Written informed consent was obtained from all participants after a full explanation of the study procedures, in accordance with REC guidelines.

## DNA isolation

Venous blood samples (3 mL) were collected from each participant into EDTA vacutainer tubes under sterile conditions. Samples were stored at − 20 °C until further analysis. Genomic DNA was extracted using the BloodZol DNA Extraction Reagent (Top-Bio, Prague, Czech Republic; Cat. No. EE131), following the manufacturer’s instructions. The concentration and purity of the extracted DNA were evaluated using the Quawell UV-Vis spectrophotometer (Q-5000).

### *Genotyping of Polymorphism “c.457T > C (rs1341667) of theSTOX1Gene”*

Genotyping of the *STOX1* gene variant (NM_001130161.3: c.457T > C; p.Tyr153His, rs1341667) was conducted using the TaqMan SNP Genotyping Assay (Thermo Fisher Scientific, Waltham, MA, USA; Cat. No. 4351379) according to the manufacturer’s instructions. PCR amplification was performed on the QuantStudio 5 Real-Time PCR System (Applied Biosystems).

The PCR thermal cycling conditions were as follows:

- Initial enzyme activation at 95 °C for 10 min to activate AmpliTaq Gold DNA polymerase.
- 40 cycles of amplification, each consisting of:
  - Denaturation at 92 °C for 15 s.
  - Annealing at 60 °C for 30 s.
  - Extension at 60 °C for 30 s.

**Fig. 1:**
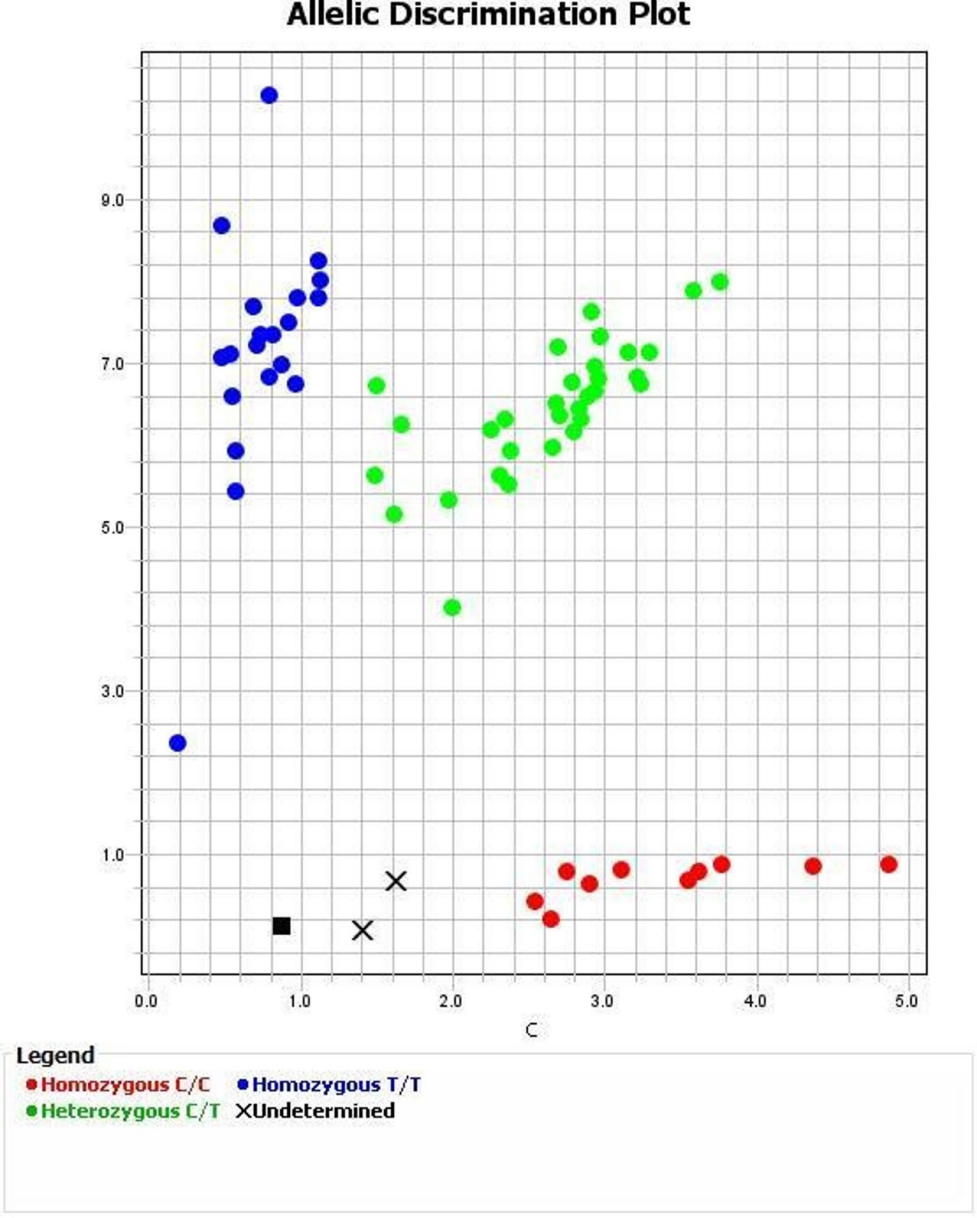
Allelic discrimination plot of STOX1 (NM_001130161.3): c.457T > C:p. (Tyr153His) by TaqMan SNP Genotyping Assay on the Applied Biosystem Step One Real-Time PCR system.

These conditions enabled specific amplification and allelic discrimination of the *STOX1* polymorphism, allowing for accurate genotyping into C/C, C/T, and T/T groups. The results of allelic discrimination are illustrated in Fig. 1.

## Statistical and genotypic association analysis

Data were analyzed using the Statistical Package for the Social Sciences (SPSS) version 28 (IBM Corp., Armonk, NY, USA). Quantitative variables were expressed as means ± standard deviations, while categorical variables were presented as frequencies and percentages. Comparisons between the case and control groups were performed using the unpaired t-test for continuous variables (Chan, 2003a) and the Chi-square (χ²) test for categorical variables. When the expected frequencies were less than 5, Fisher’s exact test was applied (Chan, 2003b). To investigate the association between the STOX1 (c.457T > C; p.Tyr153His) gene variant and the risk of preeclampsia, genotype distributions (C/C, C/T, and T/T) and allele frequencies were compared between groups using logistic regression analysis. Odds ratios (ORs) and 95% confidence intervals (CIs) were calculated, and a p-value < 0.05 was considered statistically significant. To further assess the potential association between the STOX1 c.457T > C (p.Tyr153His) variant and preeclampsia, genotype distribution was evaluated under multiple genetic inheritance models, including dominant, recessive, codominant, and overdominant models using SNPStats online tool (https://www.snpstats.net). Hardy-Weinberg equilibrium (HWE) was assessed for genotype frequencies in both case and control groups using the chi-square goodness-of-fit test. A p-value > 0.05 indicated that the observed genotype distribution did not deviate significantly from the expected distribution, confirming equilibrium. This analysis was essential for evaluating the reliability of genotyping results and ruling out potential genotyping errors, population stratification, or selection bias.

## Results

Analysis of baseline demographic characteristics (Table 1) showed that the mean gestational age at the time of sampling was significantly lower in the case group (34.15 weeks) compared to the control group (36.75 weeks; *p* < 0.001). However, this difference was not statistically associated with the distribution of either the homozygous risk genotype (CC) or the heterozygous genotype (CT) among cases with early-onset preeclampsia (Table 2). A statistically significant difference in hypertension status was also observed between the groups (*p* < 0.001), reflecting the inclusion of hypertension as a core clinical feature of preeclampsia in the case group, in contrast to the selectively normotensive control group. No other statistically significant differences were identified in the baseline demographic variables between the two groups.

**Table 1:** Demographic characteristics of preeclamptic female patients and healthy controls.

	Control	Cases	
	Mean ± Standard Deviation	Mean ± Standard Deviation	*P* value
**Age/years**	**26.73** ± **4.38**	**29.23** ± **5.52**	**0.016**
**Gestational age (weeks)**	**36.75** ± **0.44**	**34.15** ± **11.78**	**< 0.001**
**BMI**	**30.54** ± **3.40**	**31.83** ± **4.66**	**0.122**
**SBP**	**115.94** ± **8.36**	**156.52** ± **12.10**	**< 0.001**
**DBP**	**75.94** ± **7.63**	**101.02** ± **5.321**	

*: significant P value ≤ 0.05. SBP: Systolic blood pressure. DBP: diastolic blood pressure. BMI: Body Mass Index.

**Table 2:** Evaluation of genotype and the onset of preeclampsia.

		Onset				
		Early		Late		P value
		Count	%	Count	%	
**Genotype**	**Homozygous C/C**	6	31.6%	9	31.0%	0.968
	**Heterozygous C/T**	8	42.1%	11	37.9%	0.772
	**Homozygous T/T**	5	26.3%	9	31.0%	0.725

Evaluation of medical history and laboratory findings (Table 3) revealed a statistically significant difference between the two groups (*p* < 0.001), primarily due to the higher prevalence of comorbid conditions, such as hypothyroidism and a history of pregnancy related complications, including intrauterine fetal demise (IUFD), intrauterine growth restriction (IUGR), oligohydramnios, missed abortion, and placenta previa, which were reported exclusively in the case group. Urinary albumin levels were also significantly elevated in the case group. In contrast, the control group was assessed using the albumin-to-creatinine (AC) ratio, providing a more accurate measure capable of detecting traces that may be missed by dipstick testing. Both groups reported a largely negative family history of similar conditions, with the exception of a single case within the case group.

**Table 3:** Medical history and laboratory findings in both groups *: significant P value ≤ 0.05.

		Control		Cases		
		Count	%	Count	%	*P* Value
**Medical history + complications**	Yes	0	0.0%	15	31.3%	< 0.001*
	No	48	100.0%	33	68.8%	
**Family History**	+ve	0	0.0%	1	2.1%	1
	-ve	48	100.0%	47	97.9%	
**Albumin or A/C ratio**	Alb nil	48	100.0%	1	2.1%	< 0.001*
	Alb + 1	0	0.0%	11	22.9%	
	Alb + 2	0	0.0%	18	37.5%	
	Alb + 3	0	0.0%	12	25.0%	
	Alb + 4	0	0.0%	6	12.5%	

Interpretation of the genotype distribution (Table 4) showed that the variant homozygous genotype CC, previously associated with a higher risk of preeclampsia, was observed in 15 cases (31.3%) compared to 13 controls (27.1%). The heterozygous CT genotype, associated with a moderate risk, was found in 19 cases (39.6%) and 20 controls (41.7%). Both comparisons were statistically non-significant, with *p* values of 0.654 and 0.835, respectively. To further validate these findings, Hardy–Weinberg equilibrium analysis was performed (Table 5), confirming no deviation from equilibrium in the study population.

**Table 4:** Comparison of zygosity and allele frequencies between case and control Groups.

		Control (*N* = 48)		Cases (*N* = 48)		P value	OR	95% CI	
		Count	%	Count	%			Lower	Upper
**Genotype**	**Homozygous C/C**	13	27.1%	15	31.3%	0.654	1.224	0.507	2.956
	**Heterozygous C/T**	20	41.7%	19	39.6%	0.835	0.917	0.406	2.072
	**Homozygous T/T**	15	31.3%	14	29.2%	0.824	0.906	0.379	2.166
	**C/T + T/T**	35	72.9%	33	68.8%	0.654	0.817	0.338	1.974
	**allele C**	46	47.9%	49	51.0%	0.665	1.133	0.643	1.996
	**allele T**	50	52.1%	47	49.0%	0.665	0.882	0.501	1.554

Data are presented as (%), P-value ≤ 0.05 is considered significant.

**Table 5:** Hardy–Weinberg equilibrium of the genotype.

		Control		Cases	
		Count	%	Count	%
**genotype**	**Homozygous C/C**	13	27.1%	15	31.3%
	**Heterozygous C/T**	20	41.7%	19	39.6%
	**Homozygous T/T**	15	31.3%	14	29.2%
	**P**_**HW**_	0.252		0.150	

P value not significant so distribution is not deviated from Hardy Weinberg equilibrium.

The inheritance model analysis (Table 6) showed no statistically significant association between the STOX1 c.457T > C variant and preeclampsia under any of the tested models. Specifically, p-values were as follows: dominant model (*p* = 0.82), recessive model (*p* = 0.65), codominant model (*p* = 0.90), and overdominant model (*p* = 0.84). These results suggest that none of the genetic inheritance patterns of the studied variant contribute significantly to the risk of preeclampsia in the examined cohort.

**Table 6:** SNP association with response STATUS (*n* = 96, crude analysis).

Model	Genotype	STATUS = 0-Control	STATUS = 1-Case	OR (95% CI)	*P*-value	AIC	BIC
**Codominant**	T/T	15 (31.2%)	14 (29.2%)	1.00	0.9	138.9	146.6
	C/T	20 (41.7%)	19 (39.6%)	1.02 (0.39–2.66)			
	C/C	13 (27.1%)	15 (31.2%)	1.24 (0.44–3.50)			
**Dominant**	T/T	15 (31.2%)	14 (29.2%)	1.00	0.82	137	142.2
	C/T-C/C	33 (68.8%)	34 (70.8%)	1.10 (0.46–2.64)			
**Recessive**	T/T-C/T	35 (72.9%)	33 (68.8%)	1.00	0.65	136.9	142
	C/C	13 (27.1%)	15 (31.2%)	1.22 (0.51–2.96)			
**Overdominant**	T/T-C/C	28 (58.3%)	29 (60.4%)	1.00	0.84	137	142.2
	C/T	20 (41.7%)	19 (39.6%)	0.92 (0.41–2.07)			
**Log-additive**	---	---	---	1.11 (0.66–1.87)	0.69	136.9	142.1

## Discussion

Preeclampsia remains a complex, multifactorial disorder with a partially understood pathophysiological and genetic landscape. Despite numerous studies over the years, the precise genetic contributors to preeclampsia susceptibility and onset continue to be actively investigated. In the present study, we explored the association of the STOX1 (NM_001130161.3): c.457T > C; p.(Tyr153His) variant with preeclampsia risk, yet found no statistically significant relationship between this variant and either disease susceptibility or timing of onset. The distribution of both homozygous variant and heterozygous genotypes was similar between cases and controls, including among patients with early-onset preeclampsia (EOPE). These findings suggest a lack of association in the studied population.

Importantly, Hardy Weinberg equilibrium was maintained across our dataset, indicating both the absence of genotyping bias and the validity of our negative findings. Moreover, the association analysis between the SNP and preeclampsia, evaluated under different genetic inheritance models (codominant, dominant, recessive, overdominant, and log-additive), showed no statistically significant association in any model.

These results contrast with earlier studies—most notably the seminal work by Van Dijk et al. (2005), which initially identified an association between the STOX1 Y153H variant and familial preeclampsia in a Dutch cohort. That study highlighted the role of STOX1 in trophoblast differentiation, inflammatory responses, and oxidative stress pathways as possible mechanisms underlying this association.

However, our findings align more closely with several subsequent studies that failed to replicate the association between STOX1 Y153H and preeclampsia. For instance, Williams & Pipkin (2011), in a large Finnish cohort, reported no significant differences in STOX1 expression between placentas from healthy and preeclamptic women. Additionally, the variant has been broadly classified as benign^4^, further challenging its role as a pathogenic contributor.

Despite these discrepancies, interest in this variant has been recently renewed. A case–control study in the Turkish population by Pinarbasi et al.^5^ identified a significant association between Y153H and early-onset preeclampsia, although this association was not observed in late-onset cases. Similarly, a study in Colombia (LCP Niño, 2020) reported a potential link between this variant and severe forms of the disease, including those complicated by HELLP syndrome.

Our study, conducted in the Egyptian population has important limitations to consider. First, delayed diagnosis of preeclampsia in our population due to limited access to antenatal care may have led to underrepresentation of early-onset or familial cases. Second, unlike the van Dijk et al. study, we did not select participants based on family history of preeclampsia, which may have diluted any detectable genetic effect.

Although the C allele frequency was slightly higher in preeclamptic cases (51%) than in controls (47.9%), this difference was not statistically significant, echoing similar observations made by Pinarbasi et al. in their broader cohort. Another key distinction lies in phenotypic characterization: unlike the Colombian study, which focused solely on severe preeclampsia with HELLP, our study included a spectrum of disease severities, which may have introduced heterogeneity and weakened genotype–phenotype correlations.

It is also important to recognize that genetic predisposition to preeclampsia likely involves polygenic and epistatic interactions, rather than the effect of a single variant. Several recent studies have highlighted the potential combinatorial effect of the Y153H coding variant with other regulatory changes, such as the − 922 T > C promoter variant. For example, Bildirici et al. (2023) demonstrated that the presence of both variants may exert a synergistic effect on preeclampsia risk, particularly in EOPE. This underscores the importance of studying variant interactions and broader genomic context in future investigations.

Lastly, ethnic and regional genetic variability may play a critical role in disease risk. Differences observed between Turkish, Colombian, Finnish, and the studied Egyptian population may reflect underlying population-specific allele frequencies or environmental modifiers, which merit further exploration.

In conclusion, while our findings do not support an association between the STOX1 Y153H variant and preeclampsia in the studied Egyptian population, we cannot entirely rule out its contribution particularly in combination with other genetic factors. Future studies with larger, well characterized cohorts, stratification by disease severity and onset, and multi variant analyses will be essential to better define the role of STOX1 in preeclampsia pathogenesis.

## Supplementary Information

Below is the link to the electronic supplementary material.

Supplementary Material 1

Supplementary Material 2

## Data Availability

All data generated or analysed during this study are included in this published article and its supplementary information files.
