# Supplementary material for: Exploring the association between STOX1:p.(Tyr153His) variant and preeclampsia risk in Egyptian women
Source: Sci Rep. 2025 Sep 18;15:32611. doi: 10.1038/s41598-025-20238-9 (PMC12446435; doi:10.1038/s41598-025-20238-9)
Supplement: Supplementary file 1 — Supplementary Material 1 [file 41598_2025_20238_MOESM1_ESM.docx]

**Supplementary Table 1: Cases’ demographic, laboratory and clinical data**

| **No** | **Age/ years** | **Medical history of other condition** | **Gestational age /days** | **Family history** | **Preeclampsia onset *** | **Blood pressure/mmHg** | **Albumin in urine** | **BMI (Metrics Unit)** |
| --- | --- | --- | --- | --- | --- | --- | --- | --- |
| 1 | 37 | placenta previa, IUGR | 259 | Negative | Late | 150/110 | +1 | 35.9 |
| 2 | 35 | IUFD | 234 | Negative | Early | 160/100 | +1 | 45.8 |
| 3 | 33 | Missed abortion | 156 | Negative | Early | 140/80 | +2 | 33.2 |
| 4 | 31 | Null | 259 | Positive | Late | 160/110 | +2 | 31.1 |
| 5 | 21 | Null | 259 | Negative | Late | 130/80 | +1 | 29.3 |
| 6 | 22 | Missed abortion | 187 | Negative | Early | 180/120 | +2 | 29.3 |
| 7 | 20 | Null | 259 | Negative | Late | 180/ 115 | Nil | 39.1 |
| 8 | 29 | Null | 245 | Negative | Late | 160/100 | +3 | 29.3 |
| 9 | 25 | Hypothyroidism | 224 | Negative | Early | 160/110 | +2 | 44.9 |
| 10 | 21 | IUGR, Oligohydramnios | 259 | Negative | Late | 160/100 | +1 | 33.1 |
| 11 | 20 | Null | 259 | Negative | Late | 140/90 | +3 | 27.0 |
| 12 | 35 | Null | 239 | Negative | Early | 180/130 | +4 | 29.3 |
| 13 | 35 | Null | 259 | Negative | Late | 135/90 | +3 | 33.1 |
| 14 | 27 | Null | 280 | Negative | Late | 145/ 90 | +1 | 29.3 |
| 15 | 35 | Null | 259 | Negative | Late | 160/100 | +2 | 29.3 |
| 16 | 35 | Null | 259 | Negative | Late | 170/109 | +1 | 29.3 |
| 17 | 35 | Null | 237 | Negative | Early | 160/ 105 | +2 | 29.3 |
| 18 | 29 | Hypothyroidism | 235 | Negative | Early | 150/100 | +3 | 29.3 |
| 19 | 27 | Null | 259 | Negative | Late | 150/100 | +2 | 29.3 |
| 20 | 22 | Null | 259 | Negative | Late | 130/60 | +1 | 29.3 |
| 21 | 35 | Null | 259 | Negative | Late | 150/100 | +1 | 29.3 |
| 22 | 35 | Null | 251 | Negative | Late | 170/100 | +3 | 29.3 |
| 23 | 34 | Hypothyroidism | 231 | Negative | Early | 175/100 | +3 | 33.1 |
| 24 | 27 | Null | 259 | Negative | Late | 160/ 110 | +2 | 30.1 |
| 25 | 20 | Null | 253 | Negative | Late | 150/100 | +2 | 29.3 |
| 26 | 25 | Null | 259 | Negative | Late | 150/110 | +1 | 29.3 |
| 27 | 24 | Null | 259 | Negative | Late | 160/100 | +1 | 29.3 |
| 28 | 25 | Null | 205 | Negative | Early | 170/100 | +3 | 29.3 |
| 29 | 21 | Null | 174 | Negative | Early | 140/90 | +2 | 39.1 |
| 30 | 27 | Null | 252 | Negative | Late | 168/110 | +2 | 39.1 |
| 31 | 27 | IUFD | 183 | Negative | Early | 160/ 100 | +3 | 29.3 |
| 32 | 33 | Null | 209 | Negative | Early | 170/120 | +3 | 33.6 |
| 33 | 35 | Null | 259 | Negative | Late | 140/100 | +2 | 29.3 |
| 34 | 33 | Null | 259 | Negative | Late | 160/110 | +1 | 29.3 |
| 35 | 22 | placenta previa | 225 | Negative | Early | 110/90 | +2 | 39.1 |
| 36 | 30 | Null | 179 | Negative | Early | 160/100 | +2 | 29.3 |
| 37 | 32 | Null | 253 | Negative | Late | 160/110 | +4 | 29.3 |
| 38 | 35 | IUFD | 234 | Negative | Early | 160/100 | +2 | 45.8 |
| 39 | 28 | Null | 259 | Negative | Late | 120/80 | +3 | 29.3 |
| 40 | 35 | Null | 238 | Negative | Early | 170/110 | +2 | 29.3 |
| 41 | 35 | IUFR | 259 | Negative | Late | 150/100 | +2 | 29.3 |
| 42 | 35 | placenta previa | 259 | Negative | Late | 180/100 | +3 | 29.3 |
| 43 | 35 | Null | 254 | Negative | Late | 170/100 | +4 | 33.1 |
| 44 | 25 | Accidental hemorrhage | 233 | Negative | Early | 160/110 | +3 | 29.3 |
| 45 | 35 | Null | 280 | Negative | Late | 130/90 | +2 | 33.1 |
| 46 | 32 | Null | 187 | Negative | Early | 190/120 | +2 | 29.3 |
| 47 | 24 | IUFR | 238 | Negative | Early | 160/90 | +2 | 29.3 |
| 48 | 25 | Null | 259 | Negative | Late | 170/100 | +1 | 29.3 |

*preeclampsia is classified as early onset< 34 weeks, and late onset > 34 weeks of pregnancy, IUFR: Intrauterine fetal death, IUGR: Intaruterine growth retardation.
