# Supplementary material for: Exploring the association between STOX1:p.(Tyr153His) variant and preeclampsia risk in Egyptian women
Source: Sci Rep. 2025 Sep 18;15:32611. doi: 10.1038/s41598-025-20238-9 (PMC12446435; doi:10.1038/s41598-025-20238-9)
Supplement: Supplementary file 2 — Supplementary Material 2 [file 41598_2025_20238_MOESM2_ESM.docx]

| **No.** | **Age/ years** | **Gestational age/days** | **Family history** | **BP/mmHg** | **A/C ratio** | **BMI (Metrics Unit)** |
| --- | --- | --- | --- | --- | --- | --- |
|  |  |  |  |  |  |  |
| 1c | 22 | 252 | Negative | 120/80 | 5  mg/g | 29.3 |
| 2c | 34 | 259 | Negative | 120/90 | 6  mg/g | 30.9 |
| 3c | 28 | 259 | Negative | 120/70 | 6  mg/g | 28.7 |
| 4c | 23 | 252 | Negative | 110/70 | nil | 30.5 |
| 5c | 28 | 259 | Negative | 115/75 | 5  mg/g | 32.4 |
| 6c | 26 | 259 | Negative | 120/80 | 2  mg/g | 30.8 |
| 7c | 25 | 252 | Negative | 120/80 | 2  mg/g | 25.3 |
| 8c | 33 | 259 | Negative | 110/70 | 5  mg/g | 28.2 |
| 9c | 31 | 259 | Negative | 110/90 | 5  mg/g | 29.3 |
| 10c | 20 | 259 | Negative | 130/80 | nil | 27.7 |
| 11c | 29 | 259 | Negative | 110/80 | 6  mg/g | 30.0 |
| 12c | 23 | 259 | Negative | 120/70 | 6  mg/g | 39.1 |
| 13c | 23 | 259 | Negative | 110/70 | 2  mg/g | 27.2 |
| 14c | 24 | 259 | Negative | 120/80 | 2  mg/g | 28.5 |
| 15c | 26 | 252 | Negative | 120/80 | 6  mg/g | 28.5 |
| 16c | 26 | 259 | Negative | 110/80 | 6  mg/g | 29.3 |
| 17c | 25 | 259 | Negative | 95/70 | 6  mg/g | 26.3 |
| 18c | 22 | 259 | Negative | 110/70 | 6  mg/g | 35.2 |
| 19c | 27 | 259 | Negative | 110/70 | 6  mg/g | 32.0 |
| 20c | 23 | 256 | Negative | 115/90 | 6  mg/g | 29.3 |
| 21c | 25 | 259 | Negative | 120/80 | nil | 29.3 |
| 22c | 30 | 259 | Negative | 120/80 | 1  mg/g | 35.0 |
| 23c | 30 | 259 | Negative | 100/70 | 1  mg/g | 28.6 |
| 24c | 31 | 259 | Negative | 110/70 | nil | 29.4 |
| 25 c | 24 | 259 | Negative | 120/80 | 5  mg/g | 26.0 |
| 26c | 24 | 259 | Negative | 110/70 | 5  mg/g | 26.8 |
| 27c | 31 | 259 | Negative | 110/70 | 5  mg/g | 30.0 |
| 28c | 35 | 252 | Negative | 130/80 | 5  mg/g | 28.3 |
| 29c | 23 | 259 | Negative | 120/80 | 5  mg/g | 29.3 |
| 30c | 21 | 259 | Negative | 120/80 | 6  mg/g | 27.5 |
| 31c | 27 | 252 | Negative | 120/80 | nil | 26.3 |
| 32c | 29 | 259 | Negative | 110/60 | 6  mg/g | 26.9 |
| 33c | 22 | 252 | Negative | 120/80 | 6  mg/g | 27.4 |
| 34c | 35 | 259 | Negative | 120/80 | 6  mg/g | 30.8 |
| 35c | 35 | 252 | Negative | 120/90 | 6  mg/g | 32.5 |
| 36c | 20 | 259 | Negative | 120/80 | 6  mg/g | 27.9 |
| 37c | 24 | 259 | Negative | 110/80 | 2  mg/g | 34.5 |
| 38c | 26 | 259 | Negative | 120/70 | 2  mg/g | 34.3 |
| 39c | 33 | 259 | Negative | 140/70 | nil | 33.7 |
| 40c | 27 | 259 | Negative | 100/60 | 3  mg/g | 36.6 |
| 41c | 22 | 256 | Negative | 110/70 | 4  mg/g | 37.2 |
| 42c | 25 | 259 | Negative | 110/80 | 3  mg/g | 39.1 |
| 43c | 22 | 252 | Negative | 130/80 | 3  mg/g | 31.5 |
| 44c | 29 | 259 | Negative | 110/60 | nil | 30.4 |
| 45c | 31 | 252 | Negative | 130/90 | 2  mg/g | 31.6 |
| 46c | 20 | 259 | Negative | 120/70 | nil | 26.6 |
| 47c | 31 | 259 | Negative | 110/70 | 4  mg/g | 34.3 |
| 48c | 33 | 259 | Negative | 110/70 | 4  mg/g | 32.0 |
